# HFpEF Diagnosis: A Controversial Construct in Modern Cardiology

**DOI:** 10.1016/j.jaccas.2025.105594

**Published:** 2025-10-08

**Authors:** Maria Giulia Bellicini

**Affiliations:** Cardiology Unit, Spedali Civili of Brescia, Brescia, Italy

**Keywords:** acute heart failure, echocardiography, heart failure with preserved ejection fraction, HFpEF diagnosis, valvular heart disease

Heart failure with preserved ejection fraction (HFpEF) has gained significant prominence in recent years, both in academic discourse and routine clinical practice. But as its presence in trials and guidelines expands, the internal consistency of the HFpEF construct becomes increasingly difficult to defend. This commentary addresses a central concern: Although HFpEF is widely referred to as a syndrome,^1^ the evidence supporting its existence as a distinct clinical entity is fundamentally lacking.

**Take-Home Messages**

• There is no solid evidence that HFpEF as defined by guidelines exists as a distinct clinical syndrome. The combination of preserved ejection fraction, symptoms and signs of heart failure, and isolated diastolic dysfunction—absent any mimicking conditions—remains unproven in real-world cohorts.
• Most HFpEF trial populations are diagnostically heterogeneous. Common mimics such as severe valvular disease, precapillary pulmonary hypertension, and cardiomyopathies are often not excluded, undermining the internal validity of trial findings.
• Current use of the HFpEF label promotes diagnostic complacency. It functions more as a permissive placeholder than a specific diagnosis, leading to misclassification, mistreatment, and distortion of both clinical practice and research.

Many pivotal HFpEF trials, including the CHARM-Preserved, I-PRESERVE, PEP-CHF, NEAT-HFpEF, PARAGON-HF, EMPEROR-Preserved, DELIVER, PRESERVED-HF, and DEFINE-HF, have considered patients without any objective demonstration of elevated filling pressures.^2^ Echocardiographic evidence of congestion is often absent, and, critically, no echocardiographic exclusion of alternative cardiac causes of dyspnea—so-called “mimics”—is ensured. In particular, while severe valvular disease is often listed as an exclusion criterion, echocardiography is not systematically performed, and even when done, the quality and interpretation of imaging is highly variable. This omission is striking given that severe valvular disease remains one of the most frequent causes of heart failure in the presence of preserved left ventricular ejection fraction (LVEF). Compounding this issue, the NT-proBNP (N-terminal pro–B-type natriuretic peptide) cutoffs used as sufficient inclusion criterium in many of these trials has been low—comparable to those employed to *rule out* acute heart failure in acute care—thus making them clearly inappropriate for *ruling in* the diagnosis. As a result, the populations enrolled in major HFpEF trials likely include a mixture of patients with true acute heart failure due to alternative conditions (mimics), patients without any actual heart failure, and possibly a smaller subset who might meet the formal guideline definition of HFpEF. However, in the absence of rigorous diagnostic confirmation, the actual proportion of patients with “true” HFpEF remains unknown.

In our recent cohort of 773 patients hospitalized for acute heart failure, all of whom underwent systematic transthoracic echocardiography at admission performed by cardiologists with expertise in imaging, we observed that the majority of patients with LVEF >40% actually had severe valvular dysfunction.^3^^,^^4^ Among these, the most common lesions were severe tricuspid regurgitation (137 cases, 54.3%) and severe mitral regurgitation (112 cases, 44.4%), followed by severe aortic stenosis (26 cases, 10.3%), severe aortic regurgitation (16 cases, 6.3%), and severe mitral stenosis (6 cases, 2.3%). At this stage, a detailed etiological classification is not yet available. However, no case of isolated functional tricuspid regurgitation due to presumed ventricular diastolic dysfunction was identified based on initial analysis. In all instances, tricuspid regurgitation appeared secondary to significant left-sided disease or atrial functional mechanisms.^3^ Among the remaining patients with preserved or mildly reduced ejection fraction, a substantial number exhibited alternative acute cardiac conditions such as large pericardial effusion, precapillary pulmonary hypertension, high-grade atrioventricular block or sustained ventricular tachycardia, cardiomyopathy, or end-stage renal failure. Only a small minority—<10%—lacked any of these identifiable conditions. This subset, corresponding to so-called guideline-defined HFpEF (HFmrEF [heart failure with mid-range ejection fraction] patients were also included, consistent with pivotal trials and in consideration of the known inter- and intraobserver variability of echocardiographic ejection fraction assessment), emerged as a rare entity when patients were evaluated with echocardiogram at the time of acute presentation.

So what, then, is HFpEF? As currently constructed, it appears to function more as a diagnostic placeholder than a clearly defined syndrome.^2^, ^3^, ^4^, ^5^

The consequences are not benign. Misdiagnosing patients as having HFpEF may lead to inappropriate treatment with diuretics, unnecessary fluid restrictions, long-term follow-up in heart failure clinics, and the psychological weight of an ill-defined chronic condition. It also risks undermining the integrity of research trials, where heterogeneous populations and vague inclusion criteria dilute potential findings. Beyond the scientific implications, this trend signals a broader erosion of clinical reasoning. As a consultant in a tertiary center, I often observe how noncardiologists now assume that a structurally normal heart can “decompensate,” leading to repeated, unnecessary cardiology consultations. Even more concerning is the growing habit of labeling elderly patients with dyspnea and pneumonia or other respiratory-pure conditions as having pneumonia and HFpEF, for example in discharge summaries. In this environment, HFpEF becomes a permissive and nonspecific label that discourages thorough diagnostic thinking and replaces clinical precision with narrative shortcuts.

HFpEF, in its current form, is not a diagnosis. It is a construct—and a deeply problematic one—that risks distorting both clinical care and research.

## Funding Support and Author Disclosures

The author has reported that she has no relationships relevant to the contents of this paper to disclose.
